# Integrating Oral Health into India’s Universal Immunization Programme: A Strategic Pathway Toward Universal Health Coverage

**DOI:** 10.34172/ijhpm.9847

**Published:** 2026-07-12

**Authors:** Vinay Kumar Gupta, Aayushi Aggarwal, Nishita Kankane, Seema Malhotra

**Affiliations:** ^1^Department of Public Health Dentistry, Faculty of Dental Sciences, King George’s Medical University, Lucknow, India.; ^2^Directorate of Medical and Health, Lucknow, India.

## Introduction

 Oral diseases affect an estimated 3.5 billion people worldwide, which rose from 2.5 billion in 1990, reflecting a 64% increase in disability-adjusted life years over just 25 years, making them among the most prevalent and burdensome non-communicable conditions globally.^1^ Yet, oral health remains marginal within health policy discourse, frequently siloed from mainstream health system reforms in most low- and middle-income countries (LMICs), including India.^2^ This viewpoint argues that reframing oral health as a health systems and equity challenge, rather than a clinical or dental specialty issue, opens a compelling strategic opportunity to integrate a preventive oral health package within India’s Universal Immunization Programme (UIP). While oral health integration into non-dental maternal-child and primary care platforms has been explored in other settings, the present article focuses on the Indian UIP as a distinctive policy opportunity for equitable, population-scale early-life oral health prevention.Such integration represents a foundational reform aligned with India’s commitments to universal health coverage (UHC) and the Sustainable Development Goals (SDGs). As a viewpoint, this article draws on purposively selected sources, including global burden estimates, policy documents, national surveys and programme documents, and peer-reviewed empirical and review literature, chosen for their relevance to early childhood oral health, primary care integration, and the Indian UIP context. Specific quantitative statements were drawn preferentially from major burden assessments, official World Health Organization (WHO) or Government of India documents, systematic reviews or meta-analyses, and, where higher-order evidence was unavailable, from landmark primary studies.

## Oral Health Inequalities and Social Determinants

 The persistence of oral health inequalities is best understood through the lens of the Social Determinants of Health framework. Drawing on the WHO conceptual framework for social determinants of health, oral disease burden reflects the compounding effects of structural determinants mediated through intermediary factors.^3^ Illness feeds back into the social structure, exacerbating poverty through out-of-pocket payments and productivity losses. Populations with the lowest socioeconomic status carry a disproportionate burden of dental caries while simultaneously being least likely to access care. This “cliff edge of inequality” is compounded by the Inverse Care Law.^4,5^ In most LMICs, the dominant model of oral healthcare remains treatment-dominated, individualistic, specialist-oriented, and therefore structurally ill-equipped to address population-level inequities. Families seeking dental treatment face catastrophic out-of-pocket expenditures that can precipitate poverty or deepen existing disadvantage. Addressing oral health inequity, therefore, requires a fundamental reorientation of the delivery model, which should be upstream, population-wide, and integrated within broader primary health care infrastructure.^6^

## Global Oral Health Action Plan

 The global policy architecture increasingly recognises this imperative. The 74th World Health Assembly adopted Resolution WHA74.5, endorsing the WHO Global Oral Health Action Plan to ensure access to prevention, early diagnosis, and treatment of oral diseases as integral components of UHC.^7,8^ The Action Plan’s strategic plan explicitly encompasses oral health promotion and disease prevention (Objective 2), innovative workforce reform (Objective 3), and integrated oral health care delivery (Objective 4).^9^ These commitments signal a decisive normative shift from the historically downstream, curative paradigm to an upstream, preventive orientation. The UHC framework, to provide equitable access to quality health services, provides the structural logic for this shift. Integration of oral health into existing primary health care platforms can concentrate scarce resources where the disease burden is greatest, improve accountability in service delivery, and extend the reach of preventive care to vulnerable groups who would otherwise remain entirely outside the oral healthcare system. Oral health is a foundational prerequisite for achieving SDG 3, and to pursue UHC without incorporating oral health is to leave one of the most prevalent and inequitably distributed disease burdens wholly unaddressed.

## Early Childhood Caries Burden

 Untreated dental caries in deciduous teeth ranks as the 10th most prevalent condition among the non-communicable diseases, affecting 621 million worldwide, with 46.9% of children affected with Early Childhood Caries in India.^1,10^ The global economic consequences are staggering, with direct costs exceeding $356.8 billion annually and indirect costs, including lost productivity and school absenteeism, adding $187.6 billion.^9^ Yet access to preventive oral care in early childhood remains profoundly limited, particularly in rural settings where primary health infrastructure is already stretched.^11^ The integration of oral health within India’s primary care architecture is therefore not merely a policy aspiration but a developmental necessity. Evidence from high-income settings suggests that earlier preventive dental visits may reduce later treatment utilization and costs; for example, a US Medicaid study by Savage et al reported lower subsequent dental expenditures among children receiving preventive care before age one, while a Korean cohort study by Ahn et al found early dental visits to be cost-effective and associated with lower treatment costs, particularly in more deprived regions.^12,13^ However, comparable evidence for LMICs remains limited.

## Why India’s Universal Immunization Programme?

 The question, then, is not whether India should integrate preventive oral health into primary care, but how. India’s UIP offers a uniquely powerful platform for precisely this purpose. The programme reaches over 26 million newborns annually, operating through an extensive network of community health workers, Auxiliary Nurse Midwives, and primary health centres distributed across the country’s most underserved geographies.^14^ This combination of scale, repeated early-life contact, frontline workforce reach, and institutional embedding within the public health system distinguishes the UIP from many previously described oral health integration models that were local, clinic-based, or implemented in high-income settings.^15,16^ By 2016, the UIP had reduced the proportion of zero-dose children to 10.1%, demonstrating a capacity to penetrate populations that most health interventions fail to reach.^14^ The UIP is a trusted community institution that families actively engage with, creating sustained and predictable contact points in the first year of life. The nine-to-twelve-month immunization visit, in particular, represents an opportunity for the provision of preventive oral healthcare: the first teeth are erupting, dietary practices are being established, and caregivers are highly receptive to health guidance. The conceptual alignment between the “health home”^17^ and the “dental home” concept advanced by the American Academy of Pediatric Dentistry since 2001^18^ is instructive: both emphasise that early, preventive, relationship-based care within a familiar institutional setting produces more equitable outcomes than episodic, specialist-focused care. The UIP immunization visit is, in effect, India’s existing medical home, and it can be expanded, without prohibitive cost, to serve simultaneously as a dental home for every child it reaches.

## Implementation Strategy: Basic Oral Health Package and Workforce Model

 A Basic Oral Health Package delivered during routine infant immunization contacts, to facilitate establishment of a dental home within the first year of life, could include structured parental counselling on oral hygiene and feeding practices, brief visual screening for early caries risk, and referral of children with suspected need for dental assessment. This model is consistent with the oral health care pyramid (Figure), which locates basic oral health services within the remit of non-dentist personnel in low-resource settings, and with evidence showing that trained frontline providers can contribute meaningfully to oral-health promotion, risk assessment, and referral within existing child-health platforms.^19^ To justify the notion of a strategic pathway, however, implementation should be explicitly protocol-driven: immunisation and allied frontline personnel would require brief competency-based training in screening, counselling, documentation, referral triggers, and follow-up, while screening should be linked to simple standardised referral pathways with clearly identified providers, defined criteria for referral, and feedback mechanisms so that referral completion, rather than referral generation alone, becomes a measurable implementation outcome. Large-scale adoption should proceed in phases, beginning with feasibility and pilot implementation in selected districts to assess workflow fit, acceptability, referral completion, and resource requirements before wider expansion through existing UIP structures. Given the limited India-specific evidence on integrating oral health into immunisation platforms, such phased introduction should also be accompanied by an implementation-research pathway, including intervention adaptation, feasibility testing, and subsequent hybrid effectiveness-implementation evaluation to generate context-specific evidence on adoptability, feasibility, scalability, and sustainability within routine programme delivery. Monitoring and accountability frameworks should be embedded from the outset, with oral-health indicators incorporated into existing immunization programme evaluation mechanisms, so that integration strengthens, rather than fragments, India’s primary health care architecture.

**Figure:**
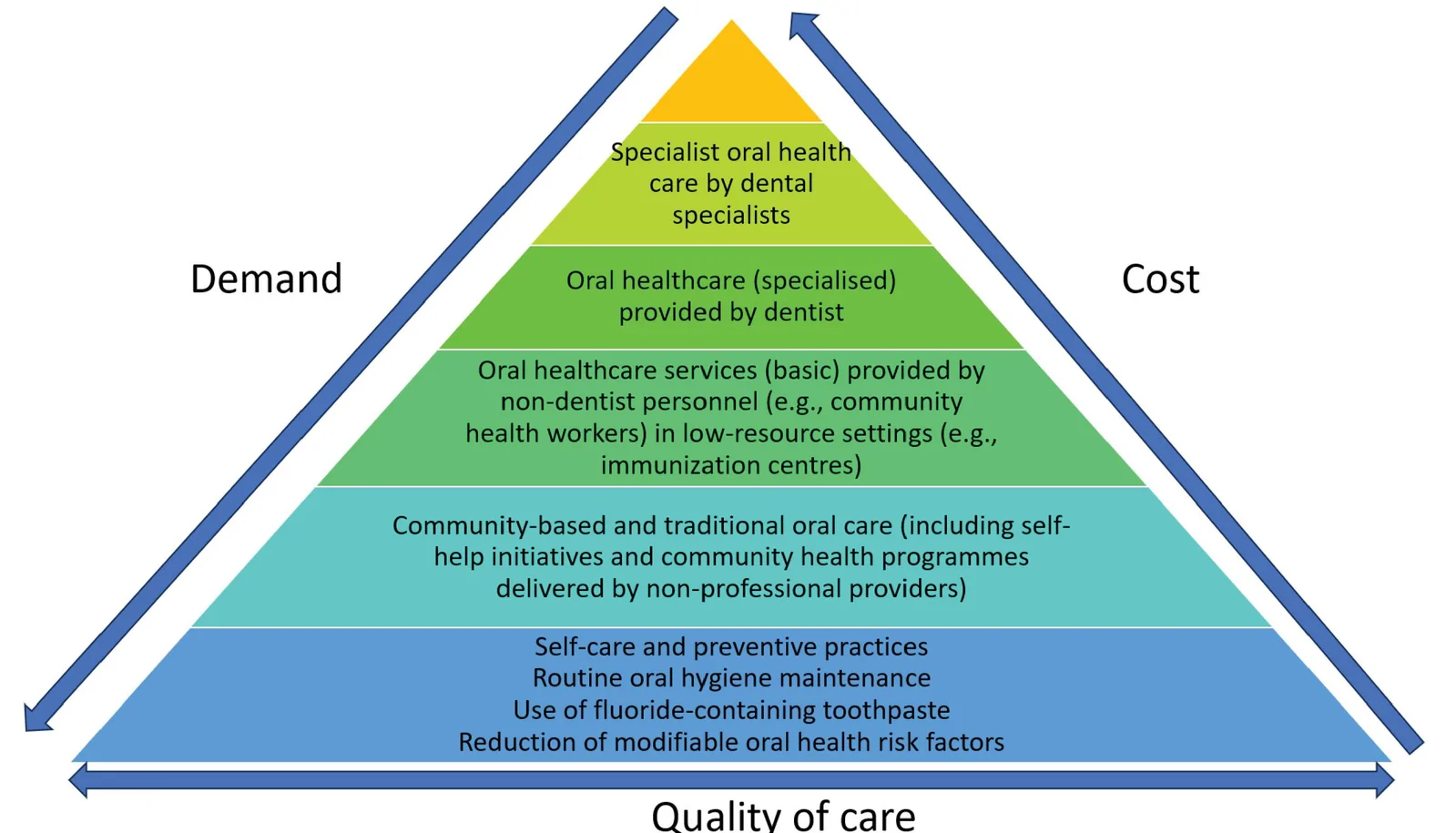
Oral healthcare in Universal Oral Health Coverage: a Focus on Providing Essential Care for the Most Common Needs With a Package of Locally Determined Cost-Effective Interventions. Adapted from Benzian and Williams.^19^

## Health Systems and Economic Implications

 From a health-systems perspective, integrating a Basic Oral Health Package within India’s UIP should be viewed as a phased service-delivery intervention. Its potential feasibility lies in using an existing national platform with repeated early-life contacts, frontline workers, outreach systems, and expanding digital infrastructure, which may make it more operationally efficient than stand-alone preventive models; however, India-specific evidence on cost-effectiveness and large-scale implementation remains limited. Wider adoption would therefore require programme-level approval, clear workforce roles, standardised protocols for counselling, screening, referral, and follow-up, and embedded monitoring through routine programme indicators. Phased pilots and implementation research are needed to establish feasibility, scalability, and economic value in the Indian context, and appraisal through appropriate mechanisms, such as Health Technology Assessment in India, would be relevant before broader policy adoption. Ongoing programme monitoring, safety oversight, and post-implementation surveillance should accompany scale-up, while any future product-based preventive components should also be subject to product-specific safety procedures.

## Policy Imperative and Call to Action

 The moment for incremental approaches has passed. Oral health is a right, not a privilege, and the persistent failure to integrate it within India’s primary health care system constitutes a structural inequity that policy must urgently correct. India’s UIP offers an opportunity to operationalise this commitment. Integrating a Basic Oral Health Package into the immunization schedule delivered by the workforce already present, at the contact point already established, is not merely a public health intervention; it is an act of health system reform consistent with India’s obligations under WHA74.5, its commitments to SDG 3, and the fundamental promise of UHC. The Ministry of Health and Family Welfare, in partnership with dental professional bodies and state health directorates, should initiate a phased implementation pathway, beginning with pilot programmes in high-burden districts and scaling through the existing UIP framework. The platform exists, and the policy rationale for pilot integration is strong, but phased evaluation is needed to establish feasibility, effectiveness, and economic value in the Indian context.

## Disclosure of artificial intelligence (AI) use

 Not applicable.

## Ethical issues

 Not applicable.

## Conflicts of interest

 Authors declare that they have no conflicts of interest.
